# Metformin Treatment and Immune Reconstitution in People With HIV and Type 2 Diabetes: A Matched Retrospective Study

**DOI:** 10.1093/ofid/ofaf110

**Published:** 2025-02-24

**Authors:** Tintin Bäckdahl, Pontus Hedberg, Jan Vesterbacka, Christina Carlander, Anders Sönnerborg, Piotr Nowak

**Affiliations:** Department of Medicine Huddinge, Karolinska Institutet, Stockholm, Sweden; Division of Infectious Diseases, Karolinska University Hospital, Stockholm, Sweden; Department of Medicine Huddinge, Karolinska Institutet, Stockholm, Sweden; Department of Medicine Huddinge, Karolinska Institutet, Stockholm, Sweden; Division of Infectious Diseases, Karolinska University Hospital, Stockholm, Sweden; Department of Medicine Huddinge, Karolinska Institutet, Stockholm, Sweden; Division of Infectious Diseases, Karolinska University Hospital, Stockholm, Sweden; Department of Medical Epidemiology and Biostatistics, Karolinska Institutet, Stockholm, Sweden; Division of Infectious Diseases, Karolinska University Hospital, Stockholm, Sweden; Department of Clinical Microbiology, Karolinska University Hospital, Stockholm, Sweden; Department of Medicine Huddinge, Karolinska Institutet, Stockholm, Sweden; Division of Infectious Diseases, Karolinska University Hospital, Stockholm, Sweden

**Keywords:** CD4, CD4/CD8, diabetes, HIV, metformin

## Abstract

**Background:**

Despite effective antiretroviral treatment (ART), HIV infection is associated with immune dysfunction and inflammation. Metformin has shown beneficial immunological and anti-inflammatory effects, including in people with HIV (PWH). We studied the potential association between metformin treatment and immune reconstitution in PWH.

**Methods:**

We conducted a retrospective cohort study set in Stockholm, Sweden. PWH with T2DM who initiated metformin treatment after at least 2 years on effective ART (exposed individuals) and metformin-naïve PWH (controls) were matched in a 1:1 ratio based on age, sex, baseline immune status, and duration of ART. Outcomes included mean values of CD4 cell counts and CD4/CD8 ratios from 1.5 years to 3.5 years after compared with 2 years before the exposed individual started metformin treatment (index date).

**Results:**

Among 1332 PWH, 43 metformin-exposed individuals (median age, 48 years; 11 years since start of ART) with T2DM and 43 nondiabetic controls (median age, 47 years; 11 years since start of ART) were included in the matched analyses. The median (interquartile range) change in CD4 T-cell count was 35 (−21 to 125) cells/μL among exposed individuals and 48 (−18 to 100) cells/μL among controls (*P* = .96). The corresponding numbers were 0.10 (0.03 to 0.20) and 0.08 (0.02–0.16) for CD4/CD8 ratio (*P* = .18). No differences were observed in subgroup analyses of PWH with low CD4 T-cell counts and CD4/CD8 ratios.

**Conclusions:**

No significant differences in immune reconstitution were observed between metformin-treated individuals and matched controls over the 2-year follow-up period.

## Teaser

We observed no significant differences in immune reconstitution (measured by CD4 T cell count and CD4/CD8 ratio) between metformin treated individuals and matched controls over a 2-year follow-up period.

Despite effective antiretroviral treatment (ART) with suppressed plasma viral levels, HIV infection is associated with immune dysfunction and persistent inflammation [1]. This contributes to progression of age-related comorbidities such as cardiovascular disease, insulin resistance and diabetes, dyslipidemia, renal impairment, lipodystrophy, and neurocognitive disorders [2]. Metformin, a widely used and cost-effective first line therapy for type 2 diabetes mellitus (T2DM), has demonstrated beneficial immunological and anti-inflammatory effects beyond its antidiabetic properties in non-HIV-infected persons [3]. This includes beneficial effects on excess weight, cardiovascular disease prevention, improvement of lipid profiles, reduction of inflammatory markers, and a reduced incidence of long COVID [4–8]. In people with HIV (PWH), metformin has been associated with immunoregulatory effects, such as reducing exhaustion markers on CD4 T cells and increasing the population of central memory CD8 T cells, which is crucial for eliminating virally infected cells [9]. However, a pilot clinical trial including 22 nondiabetic PWH showed no significant change in absolute CD4 T-cell counts or CD4/CD8 T-cell ratios between baseline, week 12, and week 24 [10]. To our knowledge, data on the long-term association between metformin and CD4 T-cell count or the CD4/CD8 ratio in PWH is lacking. Consequently, we aimed to investigate whether 2 years of metformin treatment was associated with superior reconstitution of CD4 T-cell counts and CD4/CD8 ratios in PWH.

## METHODS

### Study Population and Data Sources

We conducted a retrospective cohort study including PWH who were born between 1961 and 1981 and followed at the Department of Infectious Diseases, Karolinska University Hospital (KUH), in Stockholm, Sweden. The HIV clinic is the largest in Sweden, providing care to >2200 PWH each year. We selected a younger birth cohort (1961–1981) in an attempt to reduce factors that could potentially impact immune reconstitution and outcome of the study, such as comorbidities, other medical treatments, older ART regimes, and age-related immune dysfunction. Data from the Swedish national quality registry for HIV (InfCareHIV) and electronic medical records (EMRs) were used in the study [11]. Manual review of EMRs was used to collect information on variables not recorded in InfCareHIV. This included, for example, metformin treatment (in the treatment module of the EMRs) and diabetes status (ICD-10 codes registered in the EMRs).

### Exposure

The exposure of interest was treatment with metformin. To be considered exposed in this study, the following criteria had to be met: (i) metformin started by June 30, 2020 (to have enough follow-up data), (ii) treated with ART for at least 2 years before the start date of metformin treatment (to separate the effect of ART on immune reconstitution from possible effect of metformin), and (iii) demonstrated adherence to metformin (defined as documented compliance in medical records or decline in HbA1c after metformin initiation; nonadherence was defined as documented noncompliance in medical records or increase in HbA1c levels after metformin initiation). The date when the exposed individuals started metformin treatment will henceforward be referred to as the index date.

### Matched Cohorts

We matched PWH treated with metformin according to the criteria listed in the “Exposure” section with PWH not treated with metformin 1-to-1 without replacement. This was done by first matching all possible controls to each of the exposed individuals on the following characteristics: sex (male or female), birth year (±7 years), years since start of ART to index date (2, 3–4, 5–9, or ≥10). For the exposed individuals and the remaining eligible controls, matching was then performed based on nadir CD4 cell count, baseline CD4 cell count, and baseline CD4/CD8 ratio. The nadir CD4 cell count was defined as the lowest CD4 cell count ever measured up until the day before the index date. Baseline measurements were defined as the mean value of all measurements from 2 years to 1 day before the index date, to account for within-subject variation, including diurnal variations, in CD4 T-cell counts and CD4/CD8 ratios [12]. Exact matching on nadir CD4 cell count (cells/μL) category (<200, 200–499, and ≥500), baseline CD4 cell count category (<200, 200–499, and ≥500), and baseline CD4/CD8 category (<1 and ≥1) was performed. After this, the nadir CD4 cell count, baseline CD4 cell count, and baseline CD4/CD8 ratio of each eligible control were compared with those of the exposed individual. In order to compare CD4 cell counts and CD4/CD8 ratios in exposed individuals and controls, only individuals with baseline measurements and outcome measurements (see the “Outcomes” section and other collected variables below) available were considered. The control with the most similar nadir CD4 cell count value was ranked as 1, followed by 2 for the control with the next most similar CD4 cell count value, and so on. A final combined ranking was then created by adding together all 3 rankings for the 3 different measurements (nadir CD4 cell count, baseline CD4 cell count, and baseline CD4/CD8 ratio). The control with the best ranking was finally selected without replacement for each exposed individual.

### Outcomes and Other Collected Variables

The mean values of CD4 cell counts and CD4/CD8 ratios from 1.5 years to 3.5 years after the index date (follow-up value) were assessed to account for within-subject variation [12]. Changes in values were also calculated by subtracting the baseline value from the follow-up value.

Data on region of birth, transmission route, date of first positive HIV serology, HIV RNA copies/mL, comorbidities at baseline, ART at baseline, body mass index (BMI) at baseline, and HbA1c at baseline (only for exposed individuals) were also collected.

### Statistical Methods

We first described the characteristics of the entire study population. Continuous variables were presented as median (interquartile range [IQR]), and categorical variables were reported as frequency (percentage). The results of the matching procedure and the baseline characteristics of the exposed individuals and controls were then presented. Absolute standardized mean differences (SMDs) were used to compare continuous variables between the 2 cohorts. To investigate whether exposed individuals and controls had a different BMI trajectory over the follow-up period, the BMI during follow-up vs BMI at baseline were compared between exposed individuals and matched controls using the Wilcoxon signed-rank test.

The trajectories of CD4 T-cell counts and CD4/CD8 ratios were then compared in exposed individuals and matched controls. We started by comparing the 2 last CD4 T-cell count and CD4/CD8 ratio measurements, respectively, during the baseline period (2 years to 1 day before the index date) in exposed individuals vs controls to understand if they already had different trajectories before treatment initiation. This was done using the Wilcoxon signed-rank test. The percentages of individuals with an increase (follow-up—baseline >0) in CD4 T-cell counts and CD4/CD8 ratios were then described. The CD4 T-cell count and CD4/CD8 ratio during follow-up compared with the baseline period were then compared between exposed individuals and matched controls. Box plots and *P* values derived from the Wilcoxon signed-rank test were presented overall, as well as in 4 subgroups: (i) individuals with a CD4 T-cell count <500 cells/μL, (ii) individuals with a CD4/CD8 ratio <1, (iii) individuals with a CD4 T-cell count ≥500 cells/μL, and (iv) individuals with a CD4/CD8 ratio ≥1.

Data on BMI were missing among 4 individuals, whereas all other variables used in the matched analyses contained complete data. An alpha level of .05 was used throughout the study. All analyses were performed using R, version 4.4.1.

## RESULTS

### Characteristics of Entire Study Population

**Figure 1.**
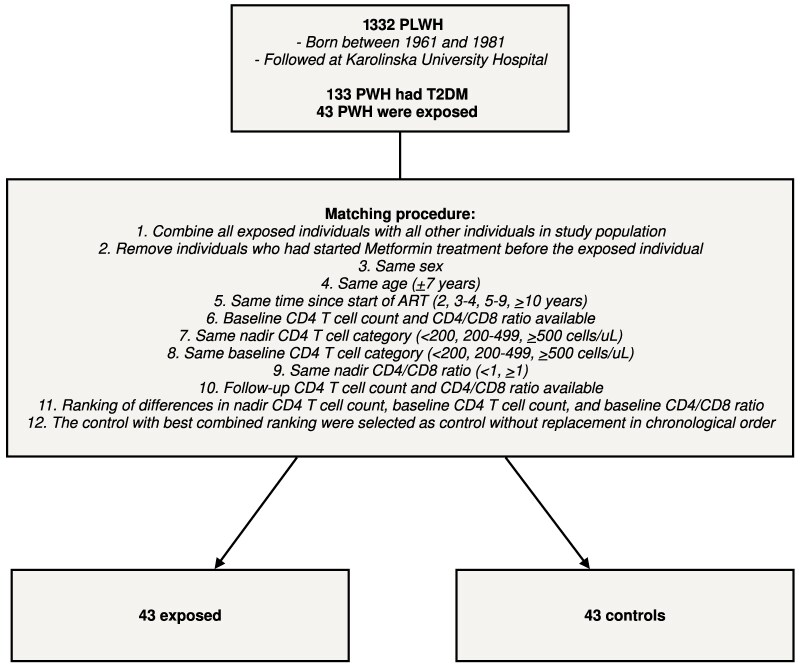
Study flowchart. Abbreviations: ART, antiretroviral treatment; PWH, people with HIV; T2DM, type 2 diabetes mellitus.

A total of 1332 PWH who were born between 1961 and 1981 and followed at Karolinska University Hospital were included in the study (Figure 1). Supplementary Table 1 presents the characteristics of the entire study population. A total of 10% (n = 133) had a T2DM diagnosis, of whom 71% (n = 95) had been treated with metformin by the end of follow-up.

### Extraction of Matched Cohorts

Of the 95 individuals who had been treated with metformin by the end of follow-up, 43 individuals were considered exposed for the matching procedure, that is, had started ART by June 2020, had been treated with ART for at least 2 years before start of metformin treatment, and showed signs of compliance during metformin treatment, defined as decline in HbA1c or documented compliance. Of the 52 metformin-treated individuals who were not considered exposed, 21 individuals had first started metformin after June 2020, 19 individuals had not been treated with ART for at least 2 years before the start of metformin treatment, and 12 individuals showed signs of limited compliance during metformin treatment. Exposed individuals were 1:1 matched to individuals who were not treated with metformin based on sex, birth year, year when ART was started, nadir CD4 cell count, baseline CD4 cell count, and baseline CD4/CD8 ratio (see the “Methods” section for more details on the matching procedure).

### Baseline Characteristics of Matched Cohorts

Baseline characteristics of the 43 exposed individuals and the 43 selected controls are presented in Table 1. All metformin-exposed individuals had diabetes mellitus, compared with no one in the control group. The metformin-exposed individuals also had a higher burden of other comorbidities, as presented in Table 1. The median (IQR) age at the index date was 48 (44–51) years among exposed individuals and 47 (43–52) years among controls (SMD, 0.16). A total of 67% (n = 29) were males in both groups. A total of 65% (n = 28) of exposed individuals were born in Sub-Saharan Africa, compared with 56% (n = 24) of controls. Heterosexual transmission was observed in 86% (n = 37) of exposed individuals and 72% (n = 31) of controls. BMI at baseline was higher in exposed individuals (median [IQR], 28 [27–32]) compared with matched controls (median [IQR], 26 [24–30]; SMD, 0.50). There was no significant change in weight during the 2-year follow-up period compared with baseline in exposed individuals vs controls (median [IQR] change, 0 [−2 to 1] kg vs 0 [−1 to 1] kg; *P* = .15).

**Table 1.** Baseline Characteristics of Exposed (Metformin-Treated) Individuals and Controls

Variable	Exposed (n = 43)	Controls (n = 43)	SMD
Age at index date, median [IQR],^a^ y	48 [44–51]	47 [43–52]	0.16
Age category at index date			
30–39 y	2 (5)	3 (7)	
40–49 y	23 (54)	24 (56)	
50–59 y	18 (42)	16 (37)	
Male sex^a^	29 (67)	29 (67)	
Region of birth			
Asia and Pacific	3 (7)	1 (2)	
Eastern Europe and Central Asia	2 (5)	2 (5)	
Latin America and the Caribbean	1 (2)	2 (5)	
Middle East and North Africa	0	3 (7)	
North America	0	0	
Sub-Saharan Africa	28 (65)	24 (56)	
Sweden	7 (16)	9 (21)	
Western Europe except Sweden	2 (5)	2 (5)	
Body mass index, median [IQR],^b^ kg/m^2^	28 [27–32]	26 [24–30]	0.50
Diabetes	43 (100)	0	
Hypertension	11 (26)	6 (14)	
Other cardiovascular diseases	3 (7)	0	
Hyperlipidemia	8 (19)	0	
Chronic kidney failure	1 (2)	1 (2)	
Liver disease	0	2 (5)	
Mental disorder	4 (9)	3 (7)	
Route of transmission			
Blood products	1 (2)	2 (5)	
Heterosexual	37 (86)	31 (72)	
Homosexual or bisexual	4 (9)	8 (19)	
Intravenous drug use	1 (2)	2 (5)	
Metformin dose, median [IQR], mg/d	1700 [1000–2000]	NA	NA
Year of first positive HIV serology, median [IQR]	2003 [1998–2009]	2003 [1997–2008]	0.08
Years since start of ART (at index date), median [IQR]	11 [7–14]	11 [8–15]	0.06
Years since start of ART category (at index date)^a^			
2	2 (5)	2 (5)	
3–4	4 (9)	4 (9)	
5–9	12 (28)	12 (28)	
≥10	25 (58)	25 (58)	
Nadir CD4 cell count, median [IQR],^a^ cells/mm^3^	160 [97–252]	160 [90–251]	0.02
Nadir CD4 cell count category^a^			
<200 cells/mm^3^	25 (58)	25 (58)	
200–499 cells/mm^3^	16 (37)	16 (37)	
≥500 cells/mm^3^	2 (5)	2 (5)	
Baseline CD4 cell count, median [IQR],^a^ cells/mm^3^	605 [506–763]	580 [501–724]	0.12
Baseline CD4 cell count category^a^			
<200 cells/mm^3^	2 (5)	2 (5)	
200–499 cells/mm^3^	9 (21)	9 (21)	
≥500 cells/mm^3^	32 (74)	32 (74)	
Baseline CD4/CD8 ratio, median [IQR],^a^ cells/mm^3^	0.9 [0.5–1.0]	0.8 [0.5–1.0]	0.09
Baseline CD4/CD8 ratio <1,^a^ cells/mm^3^	30 (70)	30 (70)	
Baseline HIV-RNA count			
<20 copies/mL	31 (72)	31 (72)	
<200 copies/mL	37 (86)	37 (86)	
<400 copies/mL	38 (88)	39 (91)	
PI at baseline	11 (26)	14 (33)	
NNRTI at baseline	10 (23)	8 (19)	
INSTI at baseline	22 (51)	20 (47)	

Abbreviations: ART, antiretroviral treatment; INSTI, integrase strand transfer inhibitor; IQR, interquartile range; NA, not applicable; NNRTI, non-nucleoside reverse transcriptase inhibitor; PI, protease inhibitor; SMD, standardized mean difference.

^a^Variables were used for matching exposed individuals and controls.

^b^Data were missing among 4 individuals.

The median (IQR) nadir CD4 cell count at the metformin start date was 160 (97–252) cells/μL among exposed individuals and 160 (90–251) cells/μL among controls (SMD, 0.02). A total of 26% (n = 11) in both groups had a baseline CD4 cell count <500 cells/μL, 70% (n = 30) had a baseline CD4/CD8 ratio <1, and 72% had an HIV-RNA count <20 copies/mL. The median (IQR) metformin dose in exposed individuals was 1700 (1000–2000) mg daily. A total of 51% (n = 22) of exposed individuals and 47% (n = 20) of controls received integrase strand transfer inhibitor (INSTI)–based antiretroviral treatment.

### Trajectory of CD4 Cell Count and CD4/CD8 Ratios

No significant differences were observed in CD4 T-cell counts and CD4/CD8 ratios between exposed individuals and controls in the 2 years before study inclusion (*P* = .50 for CD4 T-cell count and *P* = .39 for CD4/CD8 ratio). All 43 exposed individuals and their 43 matched controls had at least 1 measurement of both CD4 cell count and CD4/CD8 ratio during the baseline period (last 2 years before start of metformin) and the follow-up period (1.5–3.5 years after start of metformin). A total of 60% (n = 26) of exposed individuals and 67% (n = 29) of matched controls had an increase in CD4 T-cell count. Among those with a baseline CD4 T-cell count <500 cells/μL, all exposed individuals (n = 11) and all but one of the matched controls (n = 10) had an increase in CD4 T-cell count. A total of 81% (n = 35) of exposed individuals and 77% (n = 33) of matched controls had an increased CD4/CD8 ratio.

**Figure 2.**
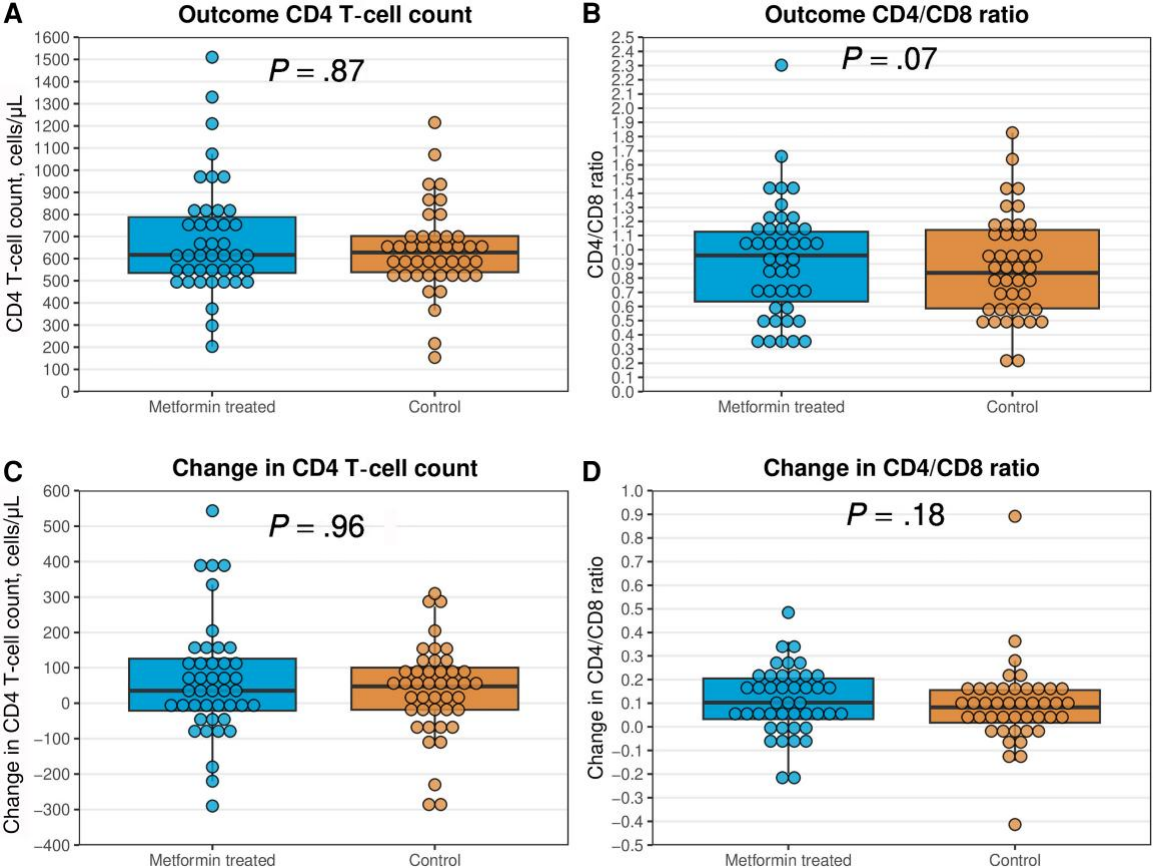
Outcome (A and B) and change (C and D) in CD4 T-cell counts and CD4/CD8 ratios in exposed individuals (n = 43) and matched controls (n = 43). The boxes represent medians with 25th and 75th percentiles. *P* values were obtained from the Wilcoxon signed-rank test. The changes in CD4 T-cell counts and CD4/CD8 ratios were obtained by calculating the average values from the outcome period (1.5–3.5 years after the index date) minus the baseline period (2 years to 1 day before the index date).

**Figure 3.**
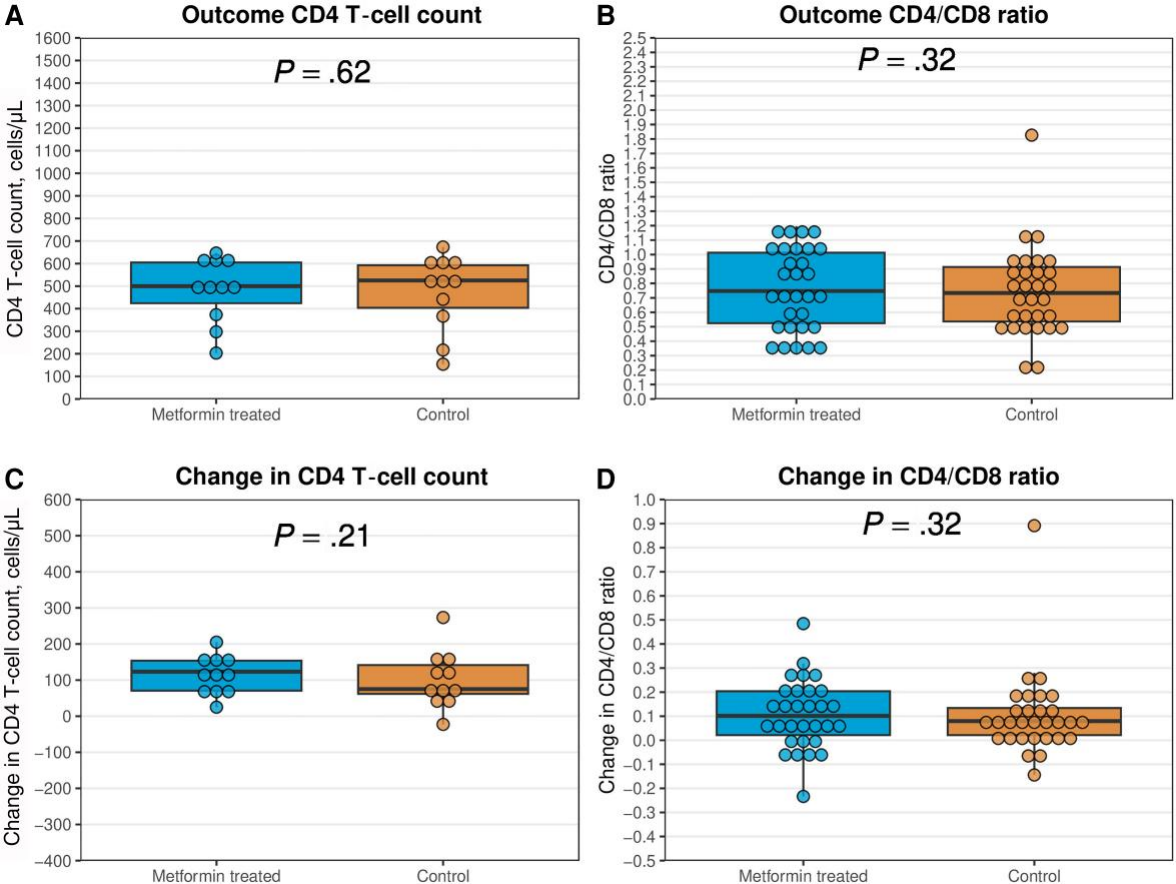
Outcome (A and B) and change (C and D) in CD4 T-cell counts and CD4/CD8 ratios in exposed individuals and matched controls with low baseline values (CD4 <500 cells/μL: 11 exposed individuals, 11 controls; CD4/CD8 <1: 30 exposed individuals, 30 controls). The boxes represent medians with 25th and 75th percentiles. *P* values were obtained from the Wilcoxon signed-rank test. The changes in CD4 T-cell counts and CD4/CD8 ratios were obtained by calculating the average values from the outcome period (1.5–3.5 years after the index date) minus the baseline period (2 years to 1 day before the index date).

The median (interquartile range) change in CD4 T-cell count was 35 (−21 to 125) cells/μL among exposed individuals and 48 (−18 to 100) cells/μL among controls (*P* = .96) (Figure 2). The corresponding numbers were 0.10 (0.03–0.20) and 0.08 (0.02–0.16) for CD4/CD8 ratio (*P* = .18). Furthermore, no differences were observed when restricting the analyses to individuals with baseline CD4 T-cell counts <500 cells/μL (11 exposed, 11 controls) and CD4/CD8 <1 (30 exposed, 30 controls) (Figure 3); neither were differences observed when restricting the analyses to those with CD4 T-cell counts ≥500 cells/μL (32 exposed, 32 controls) and CD4/CD8 < 1 (13 exposed, 13 controls) (Supplementary Figure 1).

**Figure 4.**
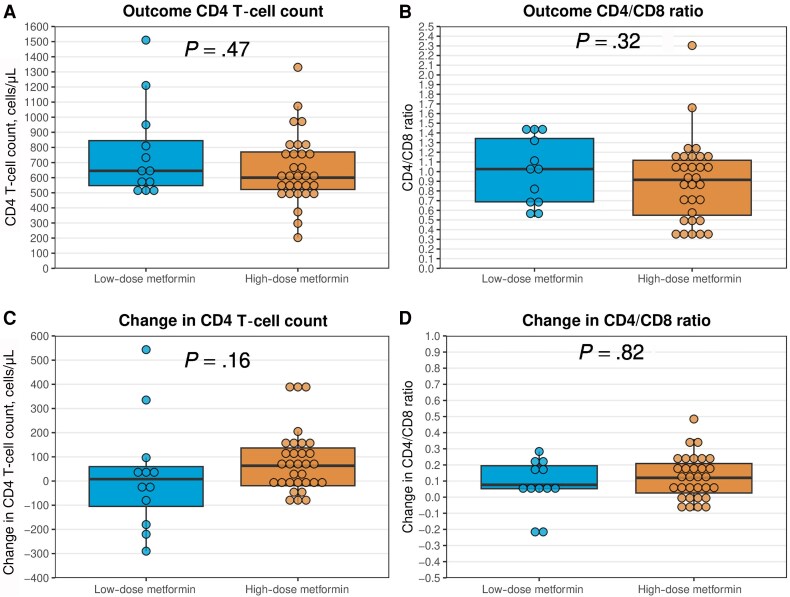
A and B, Outcome and change (C and D) in CD4 T-cell counts and CD4/CD8 ratios in exposed individuals with an effective daily dose of metformin <1000 mg (low dose, n = 12) vs ≥1000 mg (high dose, n = 31). The boxes represent medians with 25th and 75th percentiles. A dose of <1000 mg daily was characterized as low and ≥1000 mg as high. *P* values were obtained from the Wilcoxon signed-rank test. The changes in CD4 T-cell counts and CD4/CD8 ratios were obtained by calculating the average values from the outcome period (1.5–3.5 years after the index date) minus the baseline period (2 years to 1 day before the index date).

Additionally, we stratified the exposed individuals into 2 groups: those treated with a high and low daily dose of metformin. A total of 47% (n = 20) of exposed patients had dolutegravir included in their ART regimen. As dolutegravir significantly increases metformin exposure, we adjusted the metformin dose by increasing it by 60% in dolutegravir-treated patients when performing the analyses. After stratification, we found no significant difference in outcomes between patients treated with an effective daily dose of <1000 mg (n = 12) and those with an effective daily dose of ≥1000 mg (n = 31) (Figure 4).

## DISCUSSION

In this matched cohort study, we aimed to investigate whether metformin treatment was associated with the reconstitution of CD4 T-cell counts and CD4/CD8 ratios in PWH. To our knowledge, this study represents the longest follow-up and largest reporting cohort of PWH treated with metformin in this context of immune reconstitution. No difference was observed in CD4 T-cell counts or CD4/CD8 ratios between individuals treated with metformin and controls during the 2-year follow-up period. This result was consistent among all subsets of patients including individuals with lower baseline immune status (CD4 T-cell count <500 cells/μL or CD4/CD8 ratio <1).

Previous studies regarding metformin and immune reconstitution during HIV infection are scarce and have reported inconsistent results. Studies on nondiabetic PWH on ART did not find metformin treatment for 12 or 24 weeks to lead to significant changes in CD4 T-cell count, CD4/CD8 ratio, or HIV-RNA [9, 10]. On the other hand, metformin treatment in 17 PWH and T2DM was associated with more rapid immune reconstitution following the year of ART initiation, compared with 5 insulin-treated PWH in a retrospective study [13].

Several mechanisms by which metformin potentially could benefit immune reconstitution in PWH have been proposed. First, HIV infection leads to an increase in the exhaustion profile of CD4 T cells, which metformin treatment has been shown to reduce [9]. Another mechanism leading to T-cell depletion in HIV infection is gut dysbiosis, promoting mucosal damage, microbial translocation, and immune activation [14]. Metformin has been shown to enhance the diversity of microbiota in PWH, reducing local and systemic inflammation, which could in turn improve CD4 T-cell counts [15]. Additionally, metformin may exert anti-inflammatory effects by inhibiting the mechanistic target of rapamycin (mTOR), a protein kinase that controls cell metabolism and facilitates HIV transcription [10]. A 12-week treatment with metformin in nondiabetic PWH led to reduced mTOR activation in a subset of CD4 T cells and a decrease in residual HIV transcription in memory CD4 T cells in the colon. Furthermore, in a cell model, metformin increased HIV LTR promoter activity with effects on postintegration steps of the HIV-1 replication cycle, potentially resulting in decreasing viral reservoirs and HIV latency [16, 17].

We acknowledge some limitations of our work. Although efforts were made to limit differences between the matched cohorts regarding factors potentially influencing the trajectory of CD4 T-cell count and CD4/CD8 ratio, confounders may have influenced the results, the main being that metformin-exposed individuals all had T2DM and higher BMI compared with controls. Thus, immune reconstitution may be profoundly affected in individuals with T2DM, which could have influenced the study outcomes. Our cohort (although the largest reporting on PWH treated with metformin) was also limited in size, possibly leading to type II errors. Additionally, all patients had been on effective ART for a long period of time (median, 11 years) before inclusion in our study, which could have affected the potential for CD4 T-cell count gain. Nevertheless, to our knowledge, this study represents the longest follow-up on CD4 T-cell count and CD4/CD8 ratio in PWH treated with metformin.

Collectively, in our cohort study representing the longest follow-up and largest reporting cohort of PWH treated with metformin in this context, we did not observe a difference in immune reconstitution between metformin-exposed individuals and controls over a 2-year follow-up period.

## Supplementary Material

ofaf110_Supplementary_Data

## Supplementary Data

Supplementary materials are available at *Open Forum Infectious Diseases* online. Consisting of data provided by the authors to benefit the reader, the posted materials are not copyedited and are the sole responsibility of the authors, so questions or comments should be addressed to the corresponding author.
