# Supplementary material for: Metformin Treatment and Immune Reconstitution in People With HIV and Type 2 Diabetes: A Matched Retrospective Study
Source: Open Forum Infect Dis. 2025 Feb 24;12(4):ofaf110. doi: 10.1093/ofid/ofaf110 (PMC11950528; doi:10.1093/ofid/ofaf110)
Supplement: ofaf110_Supplementary_Data [file ofaf110_supplementary_data.docx]

Supplementary material for

**Metformin treatment and Immune Reconstitution in People Living with HIV and Type 2 Diabetes: A Matched Retrospective Study**

**Authors:** Tintin Bäckdahl, Pontus Hedberg, Jan Vesterbacka, Christina Carlander, Anders Sönnerborg, Piotr Nowak

Correspondence to [tintin.backdahl@ki.se](mailto:tintin.backdahl@ki.se)

**Table of contents**

| **Content** | **Page** |
| --- | --- |
| Table S1. Characteristics of the entire study population | 3 |
| Figure S1. Outcome and change in CD4 T cell counts and CD4/CD8 ratios in exposed individuals and matched controls with high baseline values (CD4 >500 cells per μL: 32 exposed individuals, 32 controls; CD4/CD8 >1: 13 exposed individuals, 13 controls) | 4 |

**Table S1. Characteristics of the entire study population**

| **Variable** | **Study population**  **(N=1,332)** |
| --- | --- |
| Year of birth, median [IQR] | 1971 [1966, 1976] |
| Male sex, n (%) | 651 (48.9) |
| Region of birth, n (%) |  |
| Asia and Pacific | 130 (9.8) |
| Eastern Europe and Central Asia | 70 (5.3) |
| Latin America and the Caribbean | 43 (3.2) |
| Middle East and North Africa | 35 (2.6) |
| North America | 6 (0.5) |
| Subsaharan Africa | 692 (52.0) |
| Sweden | 313 (23.5) |
| Western Europe except Sweden | 42 (3.2) |
| Missing | 1 (0.1) |
| Transmission route |  |
| Blood products | 23 (1.7) |
| Heterosexual | 976 (73.3) |
| Homosexual or bisexual | 185 (13.9) |
| Intravenous drug use | 94 (7.1) |
| Others or unknown | 54 (4.1) |
| Year of first positive HIV serology, median [IQR] | 2006 [2000, 2011] |
| Year of first ART | 2008 [2003, 2013] |
| Age when starting ART, years, median [IQR] | 36.0 [31.0, 42.0] |
| Nadir CD4 cell count (cells/μL), median [IQR] | 200.0 [100.0, 292.0] |
| Nadir CD4 cell count category (cells/μL) |  |
| <200 | 665 (49.9) |
| 200-499 | 580 (43.5) |
| >500 | 87 (6.5) |
| Highest CD4 cell count (cells/μL), median [IQR] | 800.0 [610.0, 1030.0] |
| Highest CD4 cell count category (cells/μL) |  |
| <200 | 7 (0.5) |
| 200-499 | 171 (12.8) |
| >500 | 1154 (86.6) |
| Nadir CD4/CD8 ratio, median [IQR] | 0.3 [0.1, 0.4] |
| Nadir CD4/CD8 ratio <1 | 1277 (95.9) |
| Highest CD4/CD8 ratio, median [IQR] | 1.1 [0.8, 1.5] |
| Highest CD4/CD8 ratio <1 | 554 (41.6) |
| Nadir HIV-RNA (copies/mL), median [IQR] | 0.0 [0.0, 0.0] |
| Nadir HIV-RNA >20 copies/mL | 17 (1.3) |
| Highest HIV-RNA (copies/mL), median [IQR] | 89150.0 [17475.0, 353250.0] |
| Highest HIV-RNA >20 copies/mL | 1284 (96.4) |
| Type 2 diabetes and metformin treatment |  |
| No type 2 diabetes | 1199 (90.0) |
| Type 2 diabetes, no metformin treatment | 38 (2.9) |
| Type 2 diabetes, metformin treatment | 95 (7.1) |

**Abbreviations:** ART=Antiretroviral treatment, IQR=Interquartile range

**Figure S1. Outcome and change in CD4 T cell counts and CD4/CD8 ratios in exposed individuals and matched controls with high baseline values (CD4 >500 cells per μL: 32 exposed individuals, 32 controls; CD4/CD8 *>*1: 13 exposed individuals, 13 controls)**

**Note:** The boxes represent the medians with the 25^th^ and 75^th^ percentiles. *P* values were obtained from Wilcoxon signed-rank tests. The change in CD4 T cell counts and CD4/CD8 ratios were obtained by calculating the average values from the outcome period (1.5 to 3.5 years after index date) minus the baseline period (2 years to 1 day before index date).
